# Clinical efficacy of denosumab, teriparatide, and oral bisphosphonates in the prevention of glucocorticoid-induced osteoporosis: a systematic review and meta-analysis

**DOI:** 10.1186/s13018-023-03920-4

**Published:** 2023-06-22

**Authors:** Chuanjian Yuan, Yanchen Liang, Kai Zhu, Wenpeng Xie

**Affiliations:** 1 Shandong University of Traditional Chinese Medicine CN, Jinan, China; 2 Affiliated Hospital of Shandong University of Traditional Chinese Medicine CN, Jinan, China

**Keywords:** Osteoporosis, Bisphosphonates, Glucocorticoid, Teriparatide, Denosumab

## Abstract

**Background:**

Continuous use of glucocorticoids (GCs) has become the primary cause of secondary osteoporosis. Bisphosphonate drugs were given priority over denosumab and teriparatide in the 2017 American College of Rheumatology (ACR) guidelines but have a series of shortcomings. This study aims to explore the efficacy and safety of teriparatide and denosumab compared with those of oral bisphosphonate drugs.

**Methods:**

We systematically searched studies included in the PubMed, Web of Science, Embase, and Cochrane library databases and included randomized controlled trials that compared denosumab or teriparatide with oral bisphosphonates. Risk estimates were pooled using both fixed and random effects models.

**Results:**

We included 10 studies involving 2923 patients who received GCs for meta-analysis, including two drug base analyses and four sensitivity analyses. Teriparatide and denosumab were superior to bisphosphonates in increasing the bone mineral density (BMD) of the lumbar vertebrae [teriparatide: mean difference [MD] 3.98%, 95% confidence interval [CI] 3.61–4.175%, *P* = 0.00001; denosumab: MD 2.07%, 95% CI 0.97–3.17%, *P* = 0.0002]. Teriparatide was superior to bisphosphonates in preventing vertebral fractures and increasing hip BMD [MD 2.39%, 95% CI 1.47–3.32, *P* < 0.00001]. There was no statistically significant difference between serious adverse events, adverse events, and nonvertebral fracture prevention drugs.

**Conclusions:**

Teriparatide and denosumab exhibited similar or even superior characteristics to bisphosphonates in our study, and we believe that they have the potential to become first-line GC-induced osteoporosis treatments, especially for patients who have previously received other anti-osteoporotic drugs with poor efficacy.

**Supplementary Information:**

The online version contains supplementary material available at 10.1186/s13018-023-03920-4.

## Background

Glucocorticoids (GCs) are a group of endogenous hormones with anti-inflammatory, immunosuppressive, and other effects and are widely used in clinical practice for the treatment of acute and chronic inflammation and a range of diseases [1]. In the Global Longitudinal Study of Osteoporosis in Women, about 2.7–4.6% of women from 10 different countries received treatment with GCs [2], and glucocorticoid-induced osteoporosis (GIOP) was the most common iatrogenic cause of secondary osteoporosis [3]. GIOP is characterized by reduced bone formation accompanied by early and transient increases in bone resorption [4]. For instance, the average incidence of fracture for those initiating use of GCs (≤ 6 months of use) was 5.1% for vertebral fracture and 2.5% for nonvertebral fracture, while the risks in chronic users of GCs (> 6 months of use) were 3.2% and 3.0%, respectively [5]. In addition, over 10% of patients on chronic GC treatment experience a clinical fracture, particularly vertebral fractures [6]. This suggests that GIOP is a major health issue that must be addressed for patients on GC medications. Bisphosphonates were given priority over denosumab and teriparatide in the American College of Rheumatology (ACR) guidelines for GIOP [7], and the efficacy of bisphosphonates in postmenopausal women and patients with GIOP is now well-recognized [8, 9]. However, oral-bisphosphonates have disadvantages such as poor patient compliance [10], poor oral absorption [11], and poor tolerability in approximately 25% of patients [12]. Teriparatide and denosumab may provide a solution to these problems.

Denosumab is a human monoclonal antibody with a high affinity for nuclear factor kappa B receptor activating ligand (RANKL), a key factor in osteoclast formation, function, and survival [13]. Denosumab can inhibit RANKL and decrease osteoclast recruitment and activity, thereby reducing bone resorption [14]. This monoclonal antibody has been reported to rapidly decrease bone resorption and increase bone mineral density (BMD) in the lumbar spine and hip, significantly reducing bone loss in postmenopausal women and in men [15]. Thus far, denosumab has been widely used for postmenopausal and other types of osteoporosis, and at the same time, it can increase bone mineral density in individuals who have used other anti-osteoporosis drugs in the past but have not benefitted from them [16].

Teriparatide is a recombinant human parathyroid hormone [17] that may be a plausible treatment for GIOP, since it directly stimulates osteoblastogenesis and inhibits osteoblast apoptosis, thereby counteracting two key mechanisms of GC therapy-promoted bone loss [18, 19].

While oral bisphosphonates are currently the most widely used bone-protective drugs in individuals on GCs, the use of teriparatide and denosumab (if approved) as first-line options in some patients merits further investigation [20]. Recent meta-analyses have also demonstrated the potential of denosumab and teriparatide as first-line treatments [21, 22]. We registered our study with PROSPERO (registration number CRD42022324526). No other systematic reviews focusing on denosumab and teriparatide vs oral bisphosphonates use for GIOP were found in the PROSPERO database.

## Methods

This systematic review and meta-analysis was performed according to the 2020 Preferred Reporting Items for Systematic Reviews and Meta-Analyses guidelines [23].

### Search strategy

**Fig. 1:**
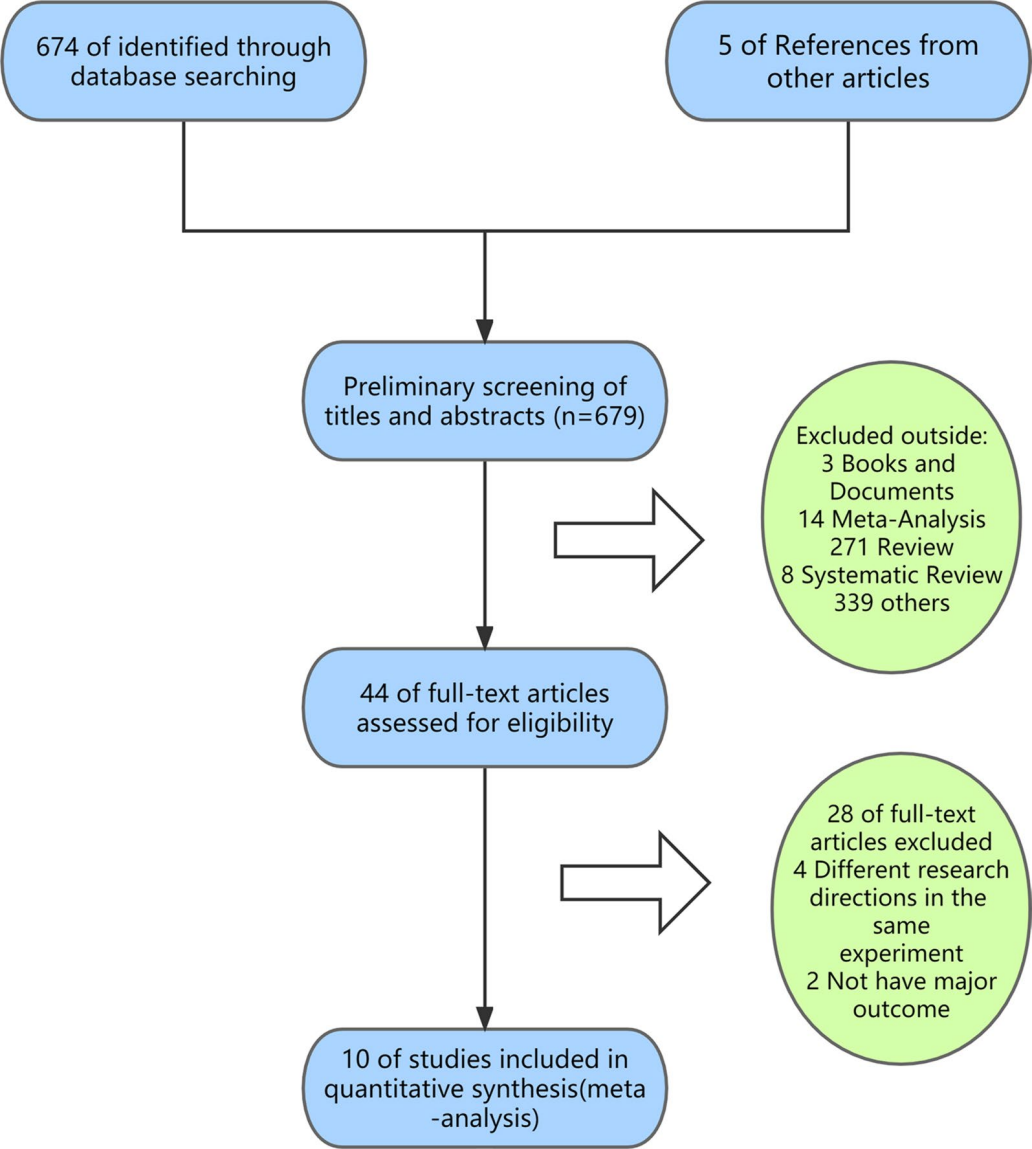
Flow diagram of literature search and study inclusion

We systematically searched for English language articles published in the PubMed, EMBASE, Cochrane, and Web of Science databases from their establishment to 5 March 2022, without sample size restriction. To retrieve all relevant articles, we used different search strategies, for example, “Osteoporosis”/”Bisphosphonates”/”Glucocorticoid,” “Osteoporosis”/”Denosumab”/”Glucocorticoid,” and “Osteoporosis”/”Teriparatide”/”Glucocorticoid.” The full search strategy used for PubMed is listed in Additional file 1. In addition, 10 articles were included based on relevant review citations. A flow diagram representing literature search and study inclusion is shown in Fig. 1.

### Study inclusion and exclusion criteria

The inclusion criteria for this study were: (1) double-blind and open-label randomized controlled trials (RCTs) lasting at least 12 months that compared either denosumab or teriparatide with one oral bisphosphonate drug; (2) studies that included patients who started or continued GC treatment and had related osteoporosis indicators. Patients starting long-term GC treatment were those who were prescribed osteoporosis drugs within 3 months of initiating GCs (i.e., primary prevention), while patients continuing long-term GC treatment were those who were prescribed osteoporosis drugs after 3 months of initiating GCs (i.e., secondary prevention) [24]; and (3) trials measuring at least one primary outcome of interest.

The exclusion criteria for this study were: (1) duplicate articles or studies with different investigations for the same population; (2) studies with patients aged < 18 years; (3) studies other than RCTs; and (4) studies including many patients with diseases or those on medications that may affect the BMD. After two of our independent searchers read the abstract and found that it was not relevant to the study of the article, or only mentioned our keywords in the abstract, we labeled such studies as “other.”

The primary outcomes of included studies involved vertebral fractures, serious adverse events, percentage change in the BMD of the lumbar spine, and secondary outcomes included nonvertebral fractures (including hip, femoral neck, and other osteoporosis-related fractures), adverse events, and percentage change in the BMD of the hip.

The pharmacological agents of interest were denosumab, teriparatide, and oral bisphosphonates (risedronate, ibandronate, and alendronate). The dosage information is shown in Tables 1 and 2. Several studies are relatively small; however, they are in line with the design principles of randomized controlled trials.

**Table 1:** Characteristics of the included studies about teriparatide

Study	Drug	N	Age (y)	Postmenopausal *n* (%)	GC dose (mg/d)	GC duration	LS BMD (gm/cm^2^) or T-score	Percentage change in lumbar spine bone	Percentage change in total hip bone
Jean-Pierre Devogelaer 2010	Teriparatide	195	55.8 ± 1.0	NM	9.4 ± 0.4	62.4 ± 7.2	− 2.51 ± 0.09 0.85 ± 0.01	6.5	3.3
	Alendronate	192	57.1 ± 1.0	NM	10.1 ± 0.7	60 ± 6	− 2.54 ± 0.07 0.85 ± 0.01	2.9	2.2
Kenneth G. Saag 2009	Teriparatide	214	56.1 ± 13.4	134 (77.9%)	7.5	18	− 2.5 ± 0.88 0.85 ± 0.13	7.2 ± 0.7	3.8 ± 0.6
	Alendronate	214	57.3 ± 14.0	143 (82.7%)	7.8	14.4	− 2.6 ± 0.89 0.85 ± 0.13	3.4 ± 0.7	2.4 ± 0.6
Alan L. 2010	Teriparatide	80	56.1 ± 2.6	41 (61%)	7.5	14.4	− 2.5 ± 0.1	NM	NM
	Alendronate	77	60.6 ± 2.5	50 (83%)	8	16.8	− 2.7 ± 0.1	NM	NM
Claus‐C. Glüer 2013	Teriparatide	45	57.5 ± 12.8	NM	8.8	85.2	− 2.48 ± 1.01	16.3 ± 4.2	NM
	Risedronate	47	55.1 ± 15.5	NM	8.8	58.8	− 2.33 ± 1.19	3.8 ± 4.1	NM
Losada 2009 Hispanic	Teriparatide	29	52.5 ± 5.0	NM	8.8 ± 1.9	63.6	0.8 ± 0.05	9.3 ± 1.7	5.9 ± 1.6
	Alendronate	32	54.9 ± 4.5	NM	7.5 ± 1.7	32.4	0.8 ± 0.05	4.4 ± 1.9	0.6 ± 1.3
Losada 2009 Nonhispanic	Teriparatide	185	58.4 ± 2.4	NM	8	5.9 ± 1.3	0.9 ± 0.02	6.5 ± 1.2	3.7 ± 0.8
	Alendronate	182	58.4 ± 2.5	NM	8	5.9 ± 1.3	0.9 ± 0.02	2.7 ± 1.2	2.7 ± 0.8
B. L. Langdahl Postmenopausal 2009	Teriparatide	134	61.9 ± 1.2	134 (100%)	7.0 (5.0, 10.0)	27.6 (6–70.8)	− 2.70 ± 0.1	7.8	NM
	Alendronate	143	62.1 ± 1.2	143 (100%)	7.3 (5.0, 10.0)	26.4 (4.8–86.4)	− 2.7 ± 0.1	3.7	NM
B. L. Langdahl Premenopausal 2009	Teriparatide	37	40.0 ± 1.9	0	8.0 (6.0, 12.5)	21.6 (4.8–68.4)	− 2.4 ± 0.2	7	NM
	Alendronate	30	35.8 ± 2.1	0	10.0 (5.0, 18.8)	10.8 (3.6–51.6)	− 2.6 ± 0.2	0.7	NM
B. L. Langdahl MAN 2009	Teriparatide	42	55.5 ± 1.9	0	10.0 (7.5, 15.0)	27.6 (8.4–68.4)	− 2.3 ± 0.2	7.3	MN
	Alendronate	41	59.7 ± 1.9	0	10.0 (5.0, 15.0)	25.2 (7.2–62.4)	− 2.3 ± 0.2	3.7	MN

**Table 2:** Characteristics of the included studies about denosumab

Study	Drug	N	Age (y)	Postmenopausal *n* (%)	GC dose (mg/d)	GC duration	LS BMD (gm/cm^2^) or T-score	Percentage change in lumbar spine bone	Percentage change in total hip bone
Ken Iseri 2017	Denosumab	14	66.5 (39.0–75.8)	5 (35.7%)	5.0 (2.4–8.5)	6.9 (2.2–19.0)	0.895 (0.745–1.060) − 1.3 (− 2.5 to − 0.3)	5.3 ± 1.1	NM
	Alendronate	14	65.5 (45.0–78.5)	4 (28.6%)	5.0 (2.5–9.3)	9.0 (1.8–19.1)	0.875 (0.821–1.045) − 1.2 (− 1.9 to 0.4)	2.0 ± 1.2	NM
Chi Chiu Mok 2021	Denosumab	69	52.0 ± 12.3	41/68 (60%)	5.1 ± 2.9	111 ± 62	0.858 ± 0.143	3.46 ± 3.0	0.9 ± 2.8
	Alendronate	70	48.0 ± 12.9	34/65 (52%)	5.0 ± 2.4	104 ± 69	0.651 ± 0.111	2.5 ± 2.9	1.5 ± 2.7
Kenneth G. Saag initiating 2018	Denosumab	253	61.5 ± 11.6	82 (88.2%)	16.6 ± 13.01	< 3	− 0.9 ± 1.9	2.8	0.9 ± 2.8
	Risedronate	252	61.3 ± 11.1	83 (89.2%)	15.6 ± 10.25	< 3	− 1.1 ± 1.6	0.8	− 0.2
Kenneth G. Saag continuing 2018	Denosumab	145	67.5 ± 10.1	159 (85.9%)	11.1 ± 7.69	> 3	− 1.9 ± 1.4	4	2.1
	Risedronate	145	64.4 ± 10.0	157 (84.9%)	12.3 ± 8.09	> 3	− 2.0 ± 1.4	2.3	0.6
Chi Chiu Mok 2015	Denosumab	21	54.9 ± 12.8	16 (76%)	4.60 ± 2.06	108.2 ± 56.0	− 2.27 ± 1.02 0.830 ± 0.11	3.39 ± 0.9	1.38 ± 0.6
	Alendronate	21	54.6 ± 13.4	14 (67%)	4.12 ± 2.14	94.1 ± 75.6	− 2.47 ± 0.99 0.810 ± 0.11	1.48 ± 0.4	0.8 ± 0.5

### Study selection and data extraction

Two reviewers independently excluded duplicate articles, and irrelevant articles were excluded by reviewing the titles and abstracts from the literature search. When one or both reviewers judged a paper as potentially eligible, its full-text version was retrieved and used for the final eligibility review. Any disagreements were resolved by discussion or the involvement of a third independent author. Data extraction from each eligible article and risk of bias assessment were performed by two reviewers who independently extracted the first author's name, publication year, study design (whether it was a double-blind RCT and other experimental designs), region of recruitment, the mean age of participants, the proportion of women and of pre- and postmenopausal women, presence or absence of basal treatment, name of the intervention and its use and dosage, the sample size of each group, and whether they had previously received medications for osteoporosis. They also extracted data on the duration of GC treatment, the dose of GC (daily dose of prednisone or equivalent during the trial), BMD of the spine (lumbar spine BMD and T-score at baseline), BMD of the hip (total hip BMD or T-score at baseline), number of vertebral and nonvertebral fractures, serious adverse events (resulting in hospitalization or withdrawal from clinical experiments), duration of adverse events, and outcome data of interest.

### Study quality assessment

#### Assessment of the risk of bias of the included studies

**Fig. 2:**
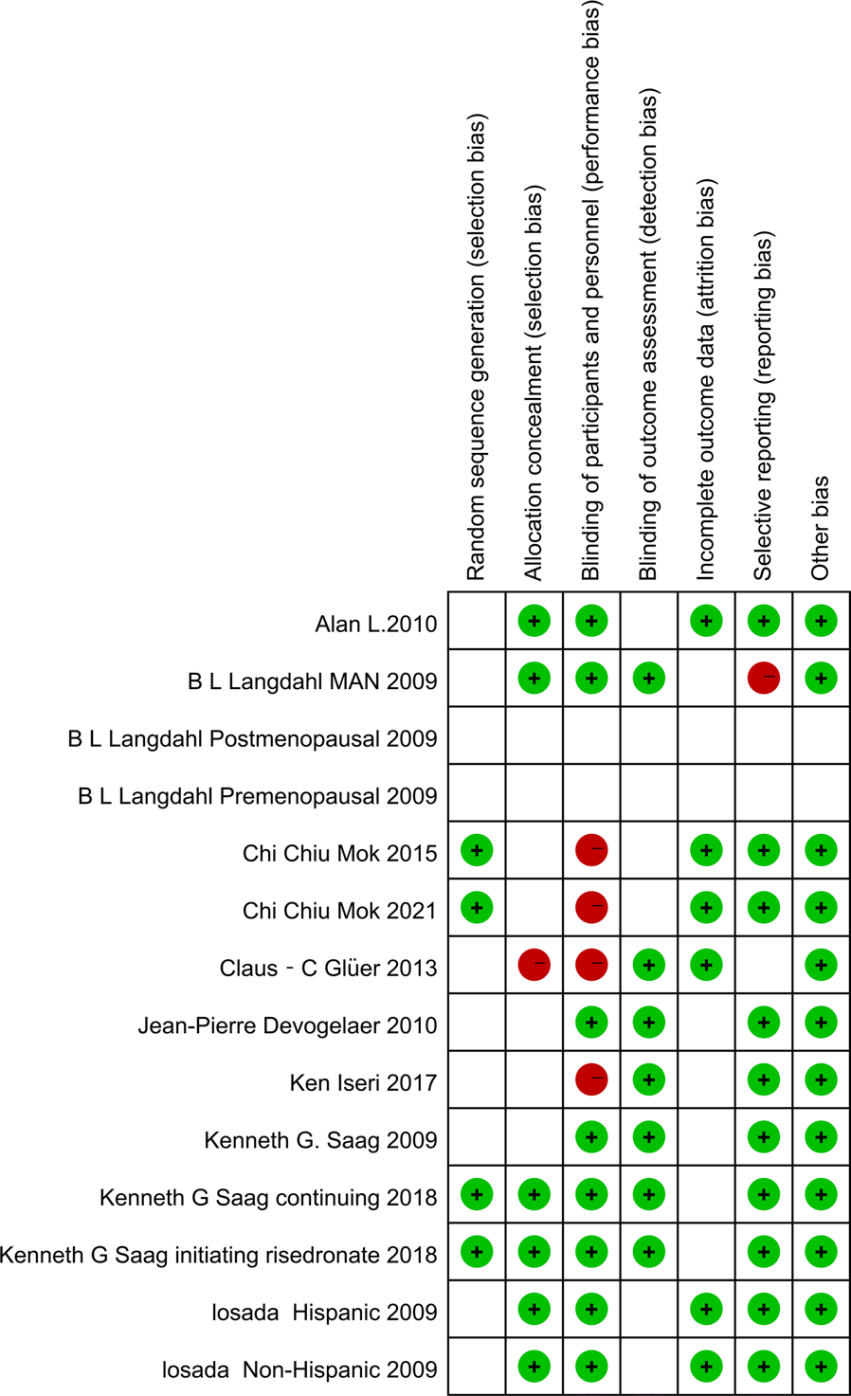
The risk figure of bias in the randomized trials

The included studies were evaluated for quality by two independent authors according to the Jadad scale. The total Jadad scoring system includes a random score, a double-blind score, and an additive combination of withdrawal and withdrawal scores. The included studies were assessed for quality, with a final score of an integer ranging from 0 to 7, and were labeled “low quality” if the score was less than 3. At the same time, we assessed the quality of the included trials with the tool used by the Cochrane collaboration to assess the risk of bias in the randomized trials (see Fig. 2) [25].

### Statistical analysis

The data were analyzed with the Review Manager 5.3 and Stata MP/16.0 software. Outcome measures results for dichotomous variables are presented as relative risk (RR) and reported with 95% confidence intervals (CIs), and for continuous type variables, mean differences (MDs) and 95% CIs were used for effect size statistical analysis. Chi-square and *I*-square (*I*^2^) tests were used to examine inter-study heterogeneity. The *I*^2^ test is the best way to measure heterogeneity between studies in a meta-analysis [26]. Furthermore, *I*^2^ values of 25%, 50%, and 75% were considered low, moderate, and high heterogeneity, respectively [27]. A fixed effects model was adopted for analysis if *I*^2^ was ≤ 50% and a sensitivity analysis was conducted if *I*^2^ was > 50%. If *I*^2^ was still > 50% after excluding marginal articles, a random effects model was used.

## Results

### Characteristics of Included Studies

Our search initially yielded 674 results, and 14 RCTs from 10 articles met the inclusion criteria [28–37] and were used for quantitative synthesis. The specific retrieval pathways are shown in Fig. 1. All but two articles [29, 33] were double-blind RCTs. The mean Jadad score was 4.6 points, with all trials scoring at least 3 points, except for two articles [29, 33] scoring 2 points. The risk of bias plots showed that the quality of the studies included in the analysis was high. The RCTs that were included were conducted within time periods ranging from 12 to 36 months, with studies in the denosumab arm all occurring within 12 months. All patients (with an average age of 52.8 years, ranged from 35.8 to 78.5 years) were given adjuvant therapy (daily calcium and vitamin D supplementation). The dose, drug duration, male-to-female ratio, and proportion of postmenopausal women are presented in Tables 1 and 2. Also, according to the study by Chiodini et al., the possible protective role of age and sex in exogenous GIOP is still partially unclear [38]. The total sample size of the 10 unique trials was 2923, and the details of each study are shown in our table.

### Network meta-analyses

#### Percentage change in the lumbar spine

**Fig. 3:**
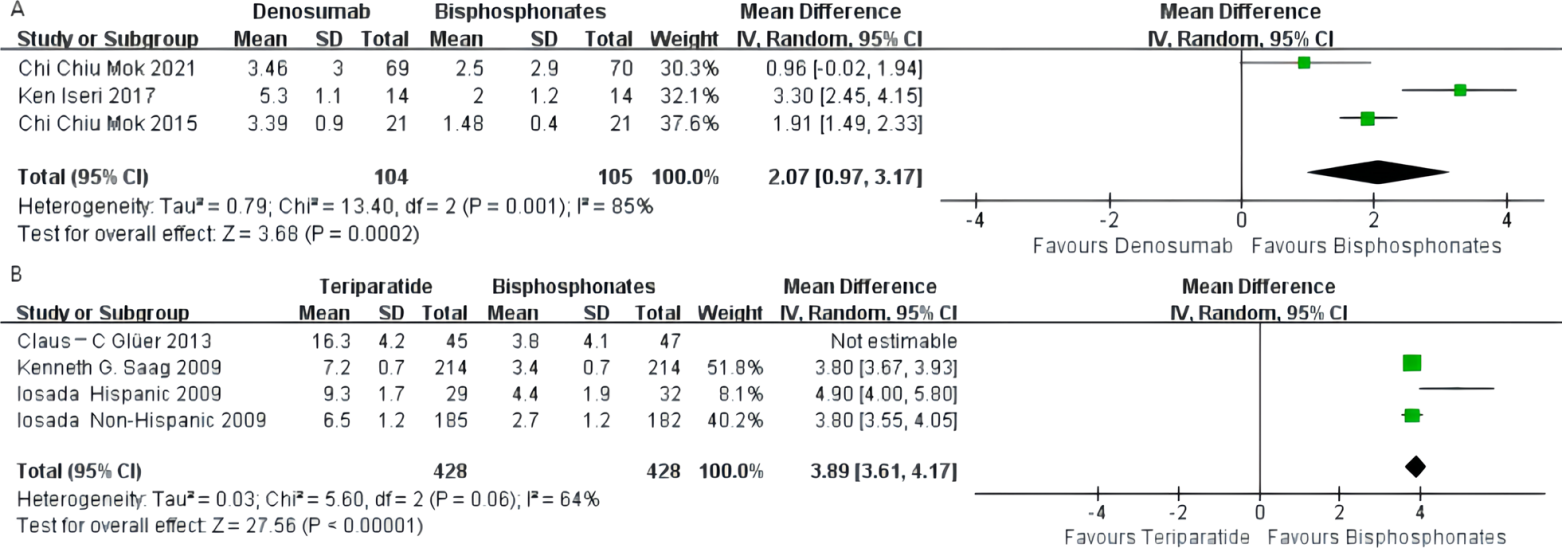
**A** Meta-analysis of denosumab-associated lumbar bone mineral density (BMD) change. **B** Meta-analysis of teriparatide-associated lumbar bone mineral density (BMD) change

The pooled results of three groups involving 209 subjects in the denosumab group indicated that denosumab was superior to oral bisphosphonates in increasing the percentage changes in the lumbar spine BMD [MD 2.07%, 95% CI 0.97–3.17%, *P* = 0.0002]. The heterogeneity test (*I*^2^ = 85%) suggested high study heterogeneity in this review. A sensitivity analysis of three publications in the denosumab group was performed to ensure data accuracy. None of these significantly interfered with the results of this meta-analysis, implying that this study had better stability and a random effects model was used for analysis (Fig. 3A).

The pooled results of the teriparatide group involving four groups of 948 subjects indicated that teriparatide was significantly better than oral bisphosphonates in increasing the percentage changes in the lumbar spine BMD. Since the heterogeneity test (*I*^2^ = 97%) suggested high study heterogeneity in this review, to ensure data accuracy, a sensitivity analysis of four RCTs in the teriparatide group was performed. The article by Glüer et al. was rejected due to high heterogeneity and a low Jadad score, and the results remained unchanged after its exclusion. Teriparatide was significantly better than bisphosphonates in increasing the lumbar spine BMD [MD 3.89%, 95% CI 3.61–4.17%, *P* = 0.00001; Fig. 3B].

### Serious adverse events

**Fig. 4:**
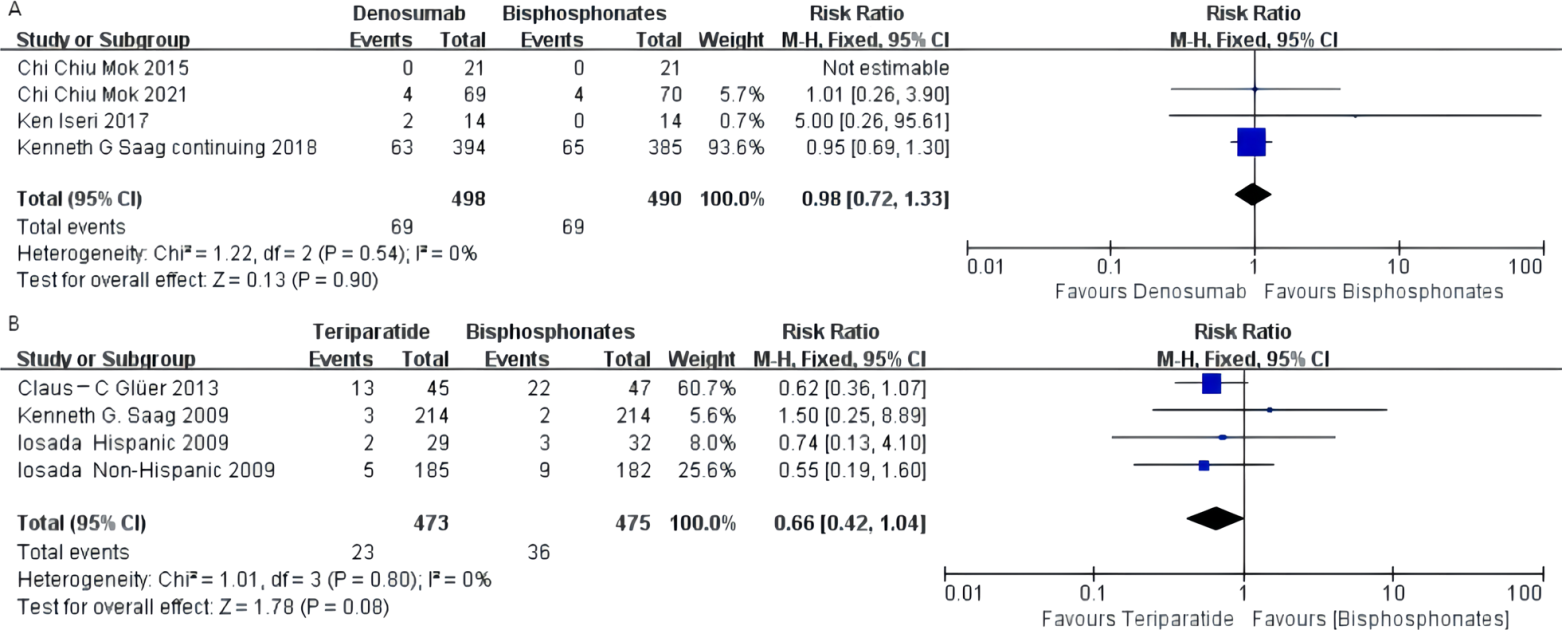
**A** Meta-analysis of denosumab-associated serious adverse events. **B** Meta-analysis of teriparatide-associated serious adverse events

Denosumab, teriparatide, and oral bisphosphonates were not significantly different regarding the incidence of serious adverse events [denosumab arm: RR 0.98, 95% CI 0.72–1.33; teriparatide arm: RR 0.66, 95% CI 0.42–1.04]. There was no statistical heterogeneity between the two groups of results (*I*^2^ = 0 in all cases). The forest plot is shown in Fig. 4A and B.

### Vertebral body fractures

**Fig. 5:**
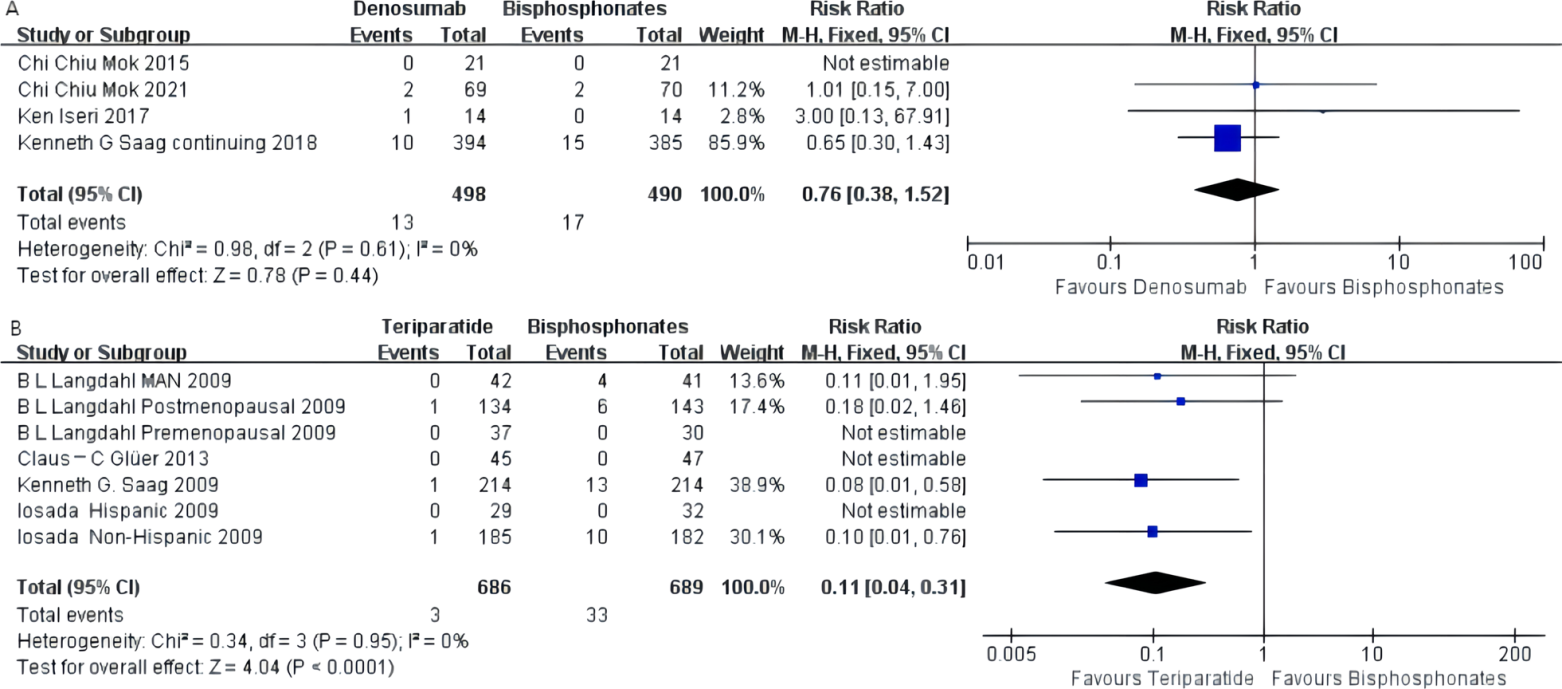
**A** Meta-analysis of denosumab-associated vertebral fractures. **B** Meta-analysis of teriparatide-associated vertebral fractures

There was no statistical difference between denosumab and oral bisphosphonates in preventing lumbar vertebral fractures [RR 0.76, 95% CI 0.38–1.52]. Heterogeneity between studies was low (*I*^2^ = 0). The forest plot is shown in Fig. 5A.

The pooled results of seven subgroups involving 1375 subjects in the teriparatide group indicated that teriparatide was significantly better than oral bisphosphonates in reducing new vertebral fractures [MD 0.11, 95% CI 0.04–0.31, *P* = 0.0001]. After the heterogeneity test, *I*^2^ = 0% indicated that the heterogeneity of this study was low, and the fixed effect model should be used for analysis. To ensure the accuracy of the results, a sensitivity analysis was performed, and there were no studies that had a greater impact on the results. The heterogeneity between studies was extremely low, and the forest plot is shown in Fig. 5B. A funnel plot was constructed to examine publication bias among the studies included in this meta-analysis, which was symmetrical and suggested a low risk of publication bias (Additional file 2: Fig. S1).

### Adverse events

**Fig. 6:**
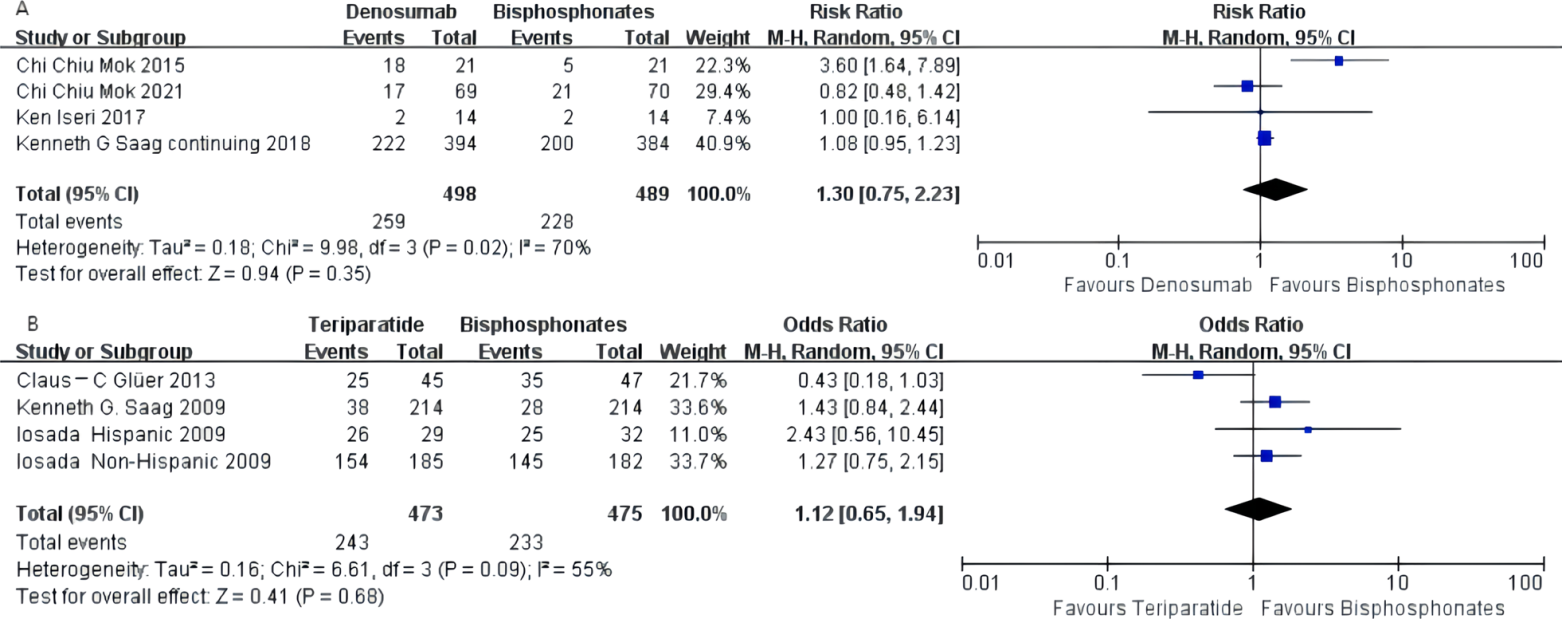
**A** Meta-analysis of denosumab-associated adverse events. **B** Meta-analysis of teriparatide-associated adverse events

Denosumab, teriparatide, and oral bisphosphonates were not significantly different regarding the incidence of adverse events [denosumab arm: RR 1.30, 95% CI 0.75–2.33; teriparatide arm: RR 1.12, 95% CI 0.65–1.94]. The results of these four subgroups were statistically heterogeneous among different studies and were analyzed using a random effects model. The forest graph is shown in Fig. 6A and B.

### New nonvertebral fractures

The lack of RCTs in the denosumab group precludes comparison.

**Fig. 7:**
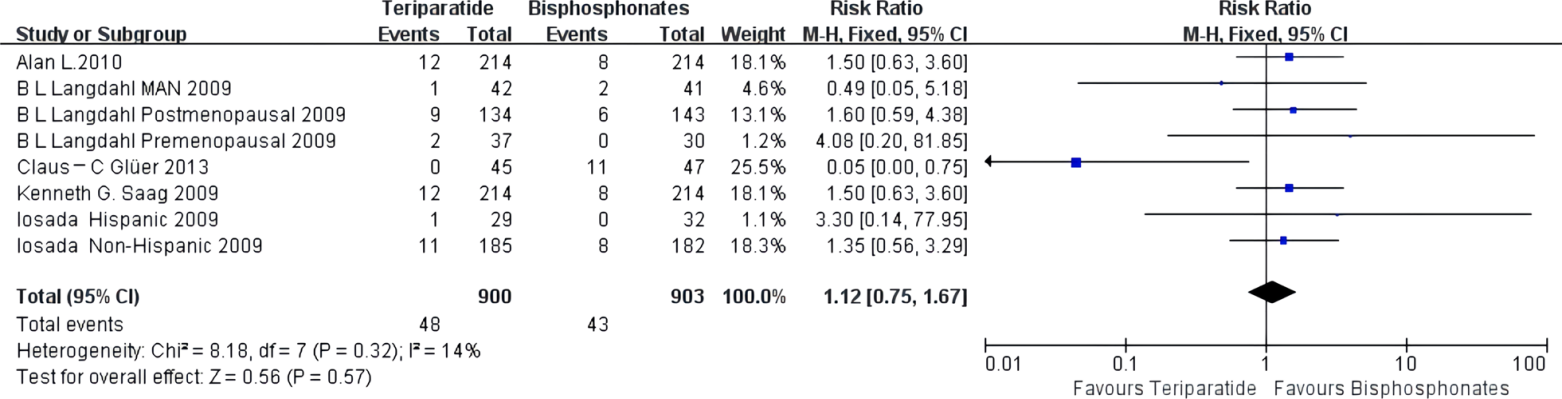
Meta-analysis of teriparatide-associated nonvertebral fractures

Teriparatide was not statistically different from oral bisphosphonates in preventing nonvertebral fractures [RR 1.12, 95% CI 0.75–1.67]. A forest plot showing low heterogeneity (*I*^2^ = 14%) among the included studies is shown in Fig. 7. Similar to vertebral fractures, a funnel plot was constructed for nonvertebral fractures as well, to examine publication bias among the studies included in this meta-analysis, which was symmetrical and suggested a low risk of publication bias (Additional file 2: Fig. S2).

### Percentage change in total hip bone

**Fig. 8:**
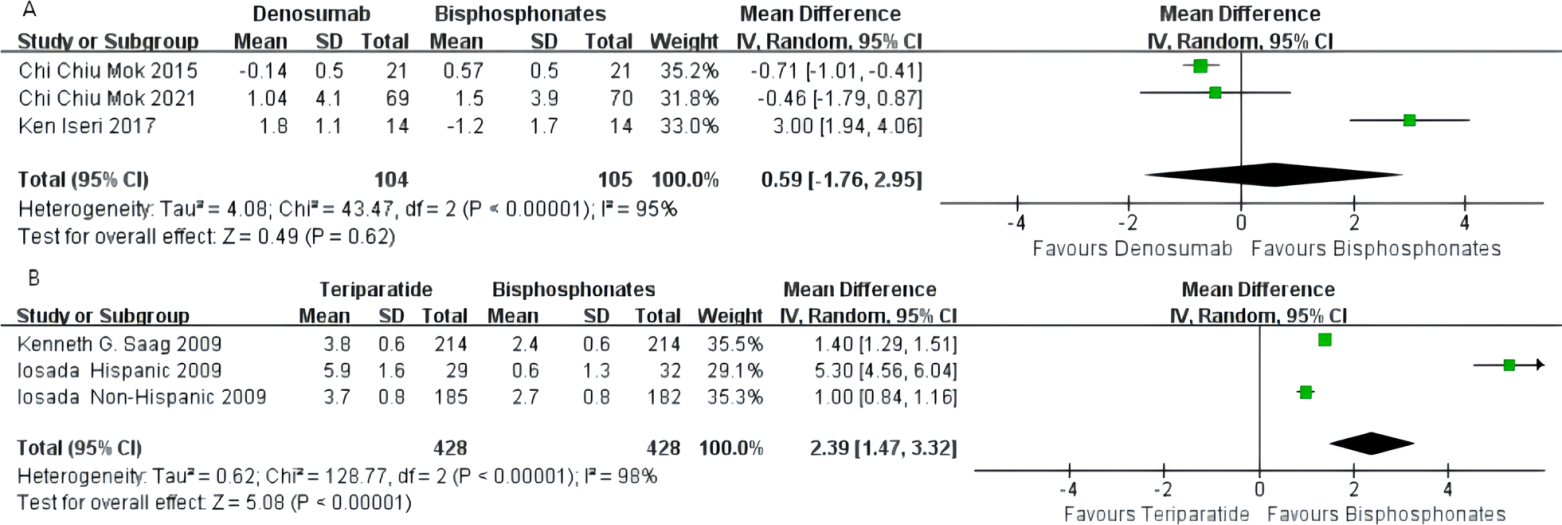
**A** Meta-analysis of denosumab-associated total hip bone mineral density (BMD) change. **B** Meta-analysis of teriparatide-associated total hip bone mineral density (BMD) change

The pooled results from three subgroups involving 209 subjects in the denosumab group indicated that oral bisphosphonates were superior to denosumab in increasing total hip BMD [MD -0.43%, 95% CI − 0.72 to 0.15%, *P* = 0.003]. The sensitivity analysis showed that rounding off any study would significantly impact the results (Additional file 2: Fig. S3). We recommend conservative acceptance of the present results, and more RCTs are needed to validate this result. The heterogeneity among different studies was high (*I*^2^ = 97%; Fig. 8A).

The pooled results of three groups involving 856 subjects showed that teriparatide was superior to oral bisphosphonates in increasing the total hip BMD [MD 2.39%, 95% CI: 1.47–3.32%, *P* < 0.00001]. The heterogeneity between studies was high (*I*^2^ = 98%; Fig. 8B).

## Discussion

Our study revealed that teriparatide and denosumab exhibited similar or even superior characteristics to bisphosphonates. To the best of our knowledge, we are the first to combine denosumab, oral bisphosphonates, and teriparatide in a direct comparison study. Unlike network meta-analysis, direct comparison results are more reliable. Based on this meta-analysis of 2923 patients, we found that teriparatide was superior to oral bisphosphonates in increasing the lumbar BMD [MD: 3.98, 95% Cl 3.61–4.17, *P* = 0.00001]. In terms of the most critical outcome (treatment for GIOP), teriparatide was superior to oral bisphosphonates in preventing vertebral fractures and increasing the hip BMD [vertebral fractures: RR 0.11, 95% CI 0.04–0.33, *P* = 0.0001; total hip BMD: MD 2.39%, 95% CI 1.47–3.32%, *P* < 0.00001], but did not differ from oral bisphosphonates in preventing nonvertebral bone fractures, which is consistent with the results of studies by Ding et al. [21], Liu et al. [22], and Liu et al. [39]. Denosumab was superior to oral bisphosphonates in increasing the lumbar spine BMD [denosumab: MD 2.07%, 95% CI 0.97–3.17%, *P* = 0.0002], and oral bisphosphonates were slightly superior to denosumab in increasing the percentage changes in total hip BMD [MD − 0.43, 95% CI − 0.72 to − 0.15%], but this result was inconsistent with the results of Zeina et al. [40]. We speculate that this is related to the low number of large RCTs included in the study; future clinical trials are still needed to prove this result, which is not significantly different from oral bisphosphonates in other respects. There was no statistically significant difference between the drugs included in the study in terms of serious and other adverse events and reduction in the risk of vertebral fractures, demonstrating that the safety profile of denosumab and teriparatide is reliable. At the same time, according to this analysis, teriparatide is superior to denosumab in some respects, which is consistent with the work of Hirooka et al. [41].

From the aspect of pathogenesis, it is now understood that cells of the osteoblast lineage are the main effectors of GC-induced bone loss and the GC-induced rise in fracture risk [42]. Thus, teriparatide seems to be the drug of first choice [5]. Direct inhibition of RANKL by denosumab should also be more effective than bisphosphonates in reducing osteocalcin levels by inhibiting osteoclast activity. In general, discontinuation of all anti-osteoporotic drugs leads to decreased BMD [43]. However, since anti-osteoporosis drugs are administered in treatment cycles, bone loss following drug withdrawal is inevitable. Therefore, all anti-osteoporosis drugs need to be treated sequentially according to clinical needs. Treatment with bisphosphonate can be performed sequentially with denosumab an inhibitor of bone resorption [44]. Teriparatide is a short-acting biological preparation, which is currently approved for 2 years. After discontinuation of treatment, the BMD will decrease rapidly, and it is recommended that treatment should be continued with bisphosphonates or denosumab to maintain the benefits of the treatment [45, 46]. Nonetheless, it is noteworthy that discontinuation of denosumab results in a significant increase in bone turnover markers and the risk of BMD loss and vertebral fractures, and it even results in a rebound in treatment efficacy [47, 48]. As suggested by Ebina et al. [49], the continuation of bisphosphonates after denosumab discontinuation may be a solution. Furthermore, teriparatide and bisphosphonates should be used with caution in fertile women [50].

From our risk of bias plot and Jadad score, the articles included in our analysis were of high quality, and those with a higher risk of bias were omitted during the study. Therefore, the credibility of our results is still relatively high. Unlike the SUCRA score, the score may differ between drugs, but the actual efficacy may not be significantly different. At the same time, we included studies with wide coverage, and the results had high general applicability.

However, this study has several limitations. Firstly, fewer RCTs were included in the denosumab versus bisphosphonates group as a whole, which meant that some of our included secondary indicators could not be meta-analyzed, and further clinical experiments are subsequently needed to verify our results. Secondly, a variety of bisphosphonates have been approved for the treatment of GIOP, including etidronate, alendronate, ibandronate, risedronate, and zoledronate [51]. Among them, alendronate, ibandronate, and risedronate are usually used as oral treatments of GIOP due to the lack of comparison between ibandronate with denosumab and teriparatide. We did not analyze them, but according to the network meta-analysis results of peers [20, 21], denosumab and teriparatide still have a degree of dominance. Thirdly, we included several studies with small sample sizes, which may be one of the reasons for the high heterogeneity. Fourthly, in terms of serious adverse events and adverse events, we made calculations based on the total number of individuals but did not analyze specific adverse events, which could be further explored in future analyses. Furthermore, bone markers might be useful to establish the optimal dose for new treatments or act as surrogate markers for fracture. Future research should focus on bone substitutes. Lastly, in accordance with Migliore et al. [52], previous fractures suggest an elevated risk of future fractures; however, due to limited data, we did not analyze the effect of previous fractures on the efficacy of the medication evaluated in this study. We also thought about including the femoral neck density and changes in the risk of atypical fractures and necrosis in the discussion, but because the RCTs included in the analysis lacked sufficient data for meta-analysis, we finally discarded them. In summary, our results still require verification with studies involving long-term treatment and follow-up with large sample sizes and more refined analysis of outcome measures and specific adverse effects. More high-quality multicenter, multiethnic clinical studies are therefore needed to validate our results.

Teriparatide and denosumab significantly increased the BMD of the lumbar spine compared with oral bisphosphonates. Teriparatide also effectively reduced the risk of lumbar spine fractures, with no apparent differences between the two drugs and oral bisphosphonates in terms of safety and tolerability. In clinical work, we suggest paying attention to the application of calcium and vitamin D. Almost all patients included in our studies received different doses of calcium and vitamin D as a base therapy, which has been shown to be effective in previous experiments [53, 54]. In the 2017 ACR guidance on GIOP, calcium and vitamin D supplementation and lifestyle changes are recommended for patients in all risk groups [7]. Furthermore, based on our previous studies, bisphosphonates are the treatment of choice for pediatric GIOP [55, 56]. Finally, in the clinic, our selection of drugs to treat GIOP is based not only on efficacy and safety, but also on its cost-effectiveness and side effects, risk factors, and mode of administration.

In conclusion, teriparatide and denosumab exhibited similar or even superior characteristics to oral bisphosphonates in our study, and we believe that they have the potential to become first-line GIOP treatments, especially for patients who have previously received other anti-osteoporotic drugs with poor efficacy.

## Supplementary Information

**Additional file 1.** Retrieval strategy of including research papers.

**Additional file 2: Figure S1** Funnel plot of publication bias in the meta-analysis of teriparatide-associated vertebral fractures. **Figure S2** Funnel plot of publication bias in the meta-analysis of teriparatide-associated nonvertebral fractures. **Figure S3** Sensitivity analysis of denosumab-associated total hip bone mineral density (BMD) change.

## Data Availability

The original contributions presented in the study are included in article/Additional file 1; further inquiries can be directed to the corresponding author.

## Abbreviations

GCs: Glucocorticoids
ACR: American College of Rheumatology
BMD: Bone mineral density
MD: Mean difference
CI: Confidence interval
GIOP: Glucocorticoid-induced osteoporosis
RCTs: Randomized controlled trials
RR: Relative risk
