# Supplementary material for: Clinical efficacy of denosumab, teriparatide, and oral bisphosphonates in the prevention of glucocorticoid-induced osteoporosis: a systematic review and meta-analysis
Source: J Orthop Surg Res. 2023 Jun 22;18:447. doi: 10.1186/s13018-023-03920-4 (PMC10286508; doi:10.1186/s13018-023-03920-4)
Supplement: Supplementary file 1 — Additional file 1. Retrieval strategy of including research papers. [file 13018_2023_3920_MOESM1_ESM.docx]

This is about the retrieval strategy of including research papers.

Supplementary Material

retrieval strategy：

1.(((((((((((((((((((((((((((((((((Bisphosphonates[Title/Abstract]) OR (Bisphosphonate[Title/Abstract])) OR (2-(Imidazol-1-yl)-1-hydroxyethylidene-1,1-bisphosphonic acid[Title/Abstract])) OR (CGP 42446A[Title/Abstract])) OR (CGP-42446[Title/Abstract])) OR (CGP 42446[Title/Abstract])) OR (CGP42446[Title/Abstract])) OR (CGP-42'446[Title/Abstract])) OR (CGP42'446[Title/Abstract])) OR (Zometa[Title/Abstract])) OR (Zoledronic Acid Anhydrous[Title/Abstract])) OR (Zoledronate[Title/Abstract])) OR (4-Amino-1-Hydroxybutylidene 1,1-Biphosphonate[Title/Abstract])) OR (Aminohydroxybutane Bisphosphonate[Title/Abstract])) OR (MK-217[Title/Abstract])) OR (MK 217[Title/Abstract])) OR (MK217[Title/Abstract])) OR (Alendronate Monosodium Salt, Trihydrate[Title/Abstract])) OR (Alendronate Sodium[Title/Abstract])) OR (Fosamax[Title/Abstract])) OR (1-Hydroxy-2-(3-pyridyl)ethylidene diphosphonate)) OR (Atelvia)) OR (Risedronate Sodium)) OR (2-(3-pyridinyl)-1-hydroxyethylidene-bisphosphonate)) OR (2-(3-pyridinyl)-1-hydroxyethylidenebisphosphonate)) OR (Risedronic Acid, Monosodium Salt)) OR (Actonel)) OR (Risedronate)) OR (Bisphosphonate Risedronate Sodium)) OR (Risedronate Sodium, Bisphosphonate)) OR (Sodium, Bisphosphonate Risedronate)) AND ((((Glucocorticoid) OR (Glucocorticoid Effect Effect, Glucocorticoid)) OR (Glucorticoid Effects)) OR (Effects, Glucorticoid))) AND ((((((((((((((((((((((((("Osteoporosis"[Mesh]) OR (Osteoporoses[Title/Abstract])) OR (Osteoporosis, Post-Traumatic[Title/Abstract])) OR (Osteoporosis, Post Traumatic[Title/Abstract])) OR (Post-Traumatic Osteoporoses[Title/Abstract])) OR (Post-Traumatic Osteoporosis[Title/Abstract])) OR (Osteoporosis, Senile[Title/Abstract])) OR (Osteoporoses, Senile[Title/Abstract])) OR (Senile Osteoporoses[Title/Abstract])) OR (Osteoporosis, Involutional[Title/Abstract])) OR (Senile Osteoporosis[Title/Abstract])) OR (Osteoporosis, Age-Related[Title/Abstract])) OR (Osteoporosis, Age Related[Title/Abstract])) OR (Bone Loss, Age-Related[Title/Abstract])) OR (Age-Related Bone Loss[Title/Abstract])) OR (Age-Related Bone Losses[Title/Abstract])) OR (Bone Loss, Age Related[Title/Abstract])) OR (Bone Losses, Age-Related[Title/Abstract])) OR (Age-Related Osteoporosis[Title/Abstract])) OR (Age Related Osteoporosis[Title/Abstract])) OR (Age-Related Osteoporoses[Title/Abstract])) OR (Osteoporoses, Age-Related[Title/Abstract])) OR (bone density[Title/Abstract])) OR (BMD[Title/Abstract]))

2.((((((Glucocorticoid) OR (Glucocorticoid Effect Effect, Glucocorticoid)) OR (Glucorticoid Effects)) OR (Effects, Glucorticoid)))) AND (((((((((((((((((((((((((("Osteoporosis"[Mesh]) OR (Osteoporoses[Title/Abstract])) OR (Osteoporosis, Post-Traumatic[Title/Abstract])) OR (Osteoporosis, Post Traumatic[Title/Abstract])) OR (Post-Traumatic Osteoporoses[Title/Abstract])) OR (Post-Traumatic Osteoporosis[Title/Abstract])) OR (Osteoporosis, Senile[Title/Abstract])) OR (Osteoporoses, Senile[Title/Abstract])) OR (Senile Osteoporoses[Title/Abstract])) OR (Osteoporosis, Involutional[Title/Abstract])) OR (Senile Osteoporosis[Title/Abstract])) OR (Osteoporosis, Age-Related[Title/Abstract])) OR (Osteoporosis, Age Related[Title/Abstract])) OR (Bone Loss, Age-Related[Title/Abstract])) OR (Age-Related Bone Loss[Title/Abstract])) OR (Age-Related Bone Losses[Title/Abstract])) OR (Bone Loss, Age Related[Title/Abstract])) OR (Bone Losses, Age-Related[Title/Abstract])) OR (Age-Related Osteoporosis[Title/Abstract])) OR (Age Related Osteoporosis[Title/Abstract])) OR (Age-Related Osteoporoses[Title/Abstract])) OR (Osteoporoses, Age-Related[Title/Abstract])) OR (bone density[Title/Abstract])) OR (BMD[Title/Abstract])))) AND (((((Denosumab[Mesh]) OR (Xgeva[Title/Abstract])) OR (AMG 162[Title/Abstract])) OR (Prolia[Title/Abstract]))

3.(((((((hPTH (1-34)) OR (Human Parathyroid Hormone (1-34))) OR (Parathar)) OR (Teriparatide Acetate)) OR (Forteo)) OR (teriparatide)) AND (((((Glucocorticoid) OR (Glucocorticoid Effect Effect, Glucocorticoid)) OR (Glucorticoid Effects)) OR (Effects, Glucorticoid))))) AND (((((((((((((((((((((((((("Osteoporosis"[Mesh]) OR (Osteoporoses[Title/Abstract])) OR (Osteoporosis, Post-Traumatic[Title/Abstract])) OR (Osteoporosis, Post Traumatic[Title/Abstract])) OR (Post-Traumatic Osteoporoses[Title/Abstract])) OR (Post-Traumatic Osteoporosis[Title/Abstract])) OR (Osteoporosis, Senile[Title/Abstract])) OR (Osteoporoses, Senile[Title/Abstract])) OR (Senile Osteoporoses[Title/Abstract])) OR (Osteoporosis, Involutional[Title/Abstract])) OR (Senile Osteoporosis[Title/Abstract])) OR (Osteoporosis, Age-Related[Title/Abstract])) OR (Osteoporosis, Age Related[Title/Abstract])) OR (Bone Loss, Age-Related[Title/Abstract])) OR (Age-Related Bone Loss[Title/Abstract])) OR (Age-Related Bone Losses[Title/Abstract])) OR (Bone Loss, Age Related[Title/Abstract])) OR (Bone Losses, Age-Related[Title/Abstract])) OR (Age-Related Osteoporosis[Title/Abstract])) OR (Age Related Osteoporosis[Title/Abstract])) OR (Age-Related Osteoporoses[Title/Abstract])) OR (Osteoporoses, Age-Related[Title/Abstract])) OR (bone density[Title/Abstract])) OR (BMD[Title/Abstract])))
