# Supplementary material for: Clinical efficacy of denosumab, teriparatide, and oral bisphosphonates in the prevention of glucocorticoid-induced osteoporosis: a systematic review and meta-analysis
Source: J Orthop Surg Res. 2023 Jun 22;18:447. doi: 10.1186/s13018-023-03920-4 (PMC10286508; doi:10.1186/s13018-023-03920-4)
Supplement: Supplementary file 2 — Additional file 2: Figure S1 Funnel plot of publication bias in the meta-analysis of teriparatide-associated vertebral fractures. Figure S2 Funnel plot of publication bias in the meta-analysis of teriparatide-associated nonvertebral fractures. Figure S3 Sensitivity analysis of denosumab-associated total hip bone mineral density (BMD) change. [file 13018_2023_3920_MOESM2_ESM.docx]

This is about the retrieval strategy of including research papers.

Supplementary Material

1.Supplementary Figures


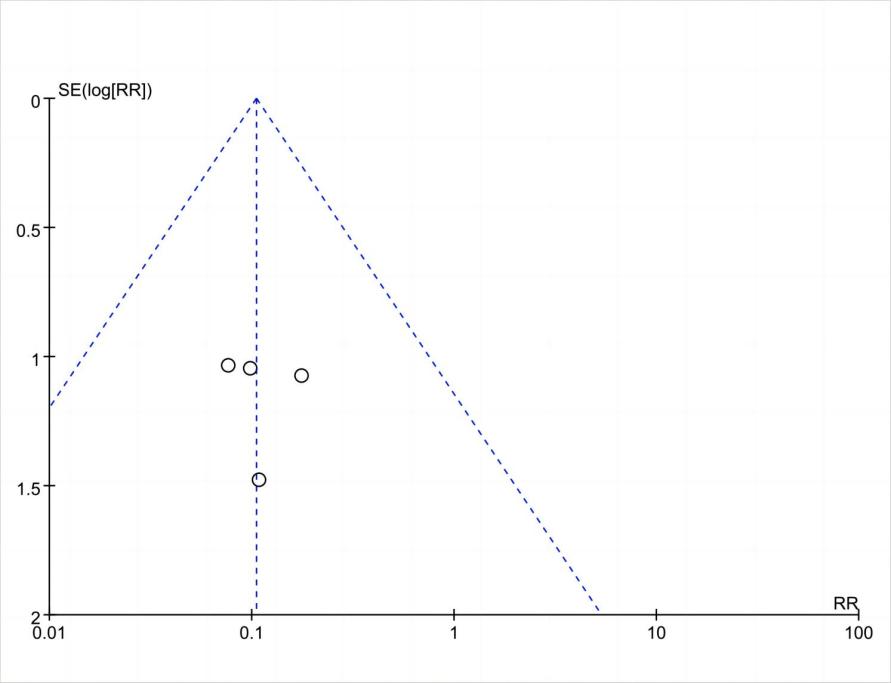


Supplementary Figure 1：Funnel plot of publication bias in the meta-analysis of teriparatide-associated vertebral fractures.


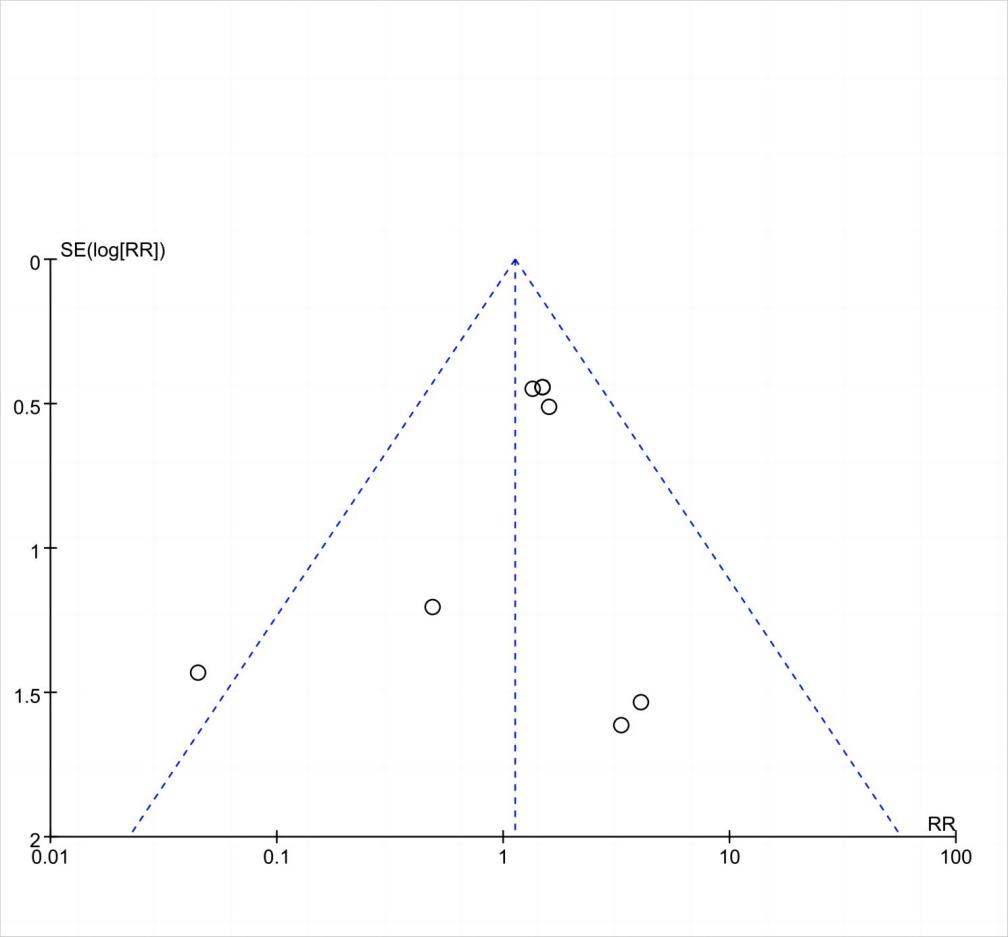


Supplementary Figure 2：Funnel plot of publication bias in the meta-analysis of Teriparatide-associated nonvertebral fractures.


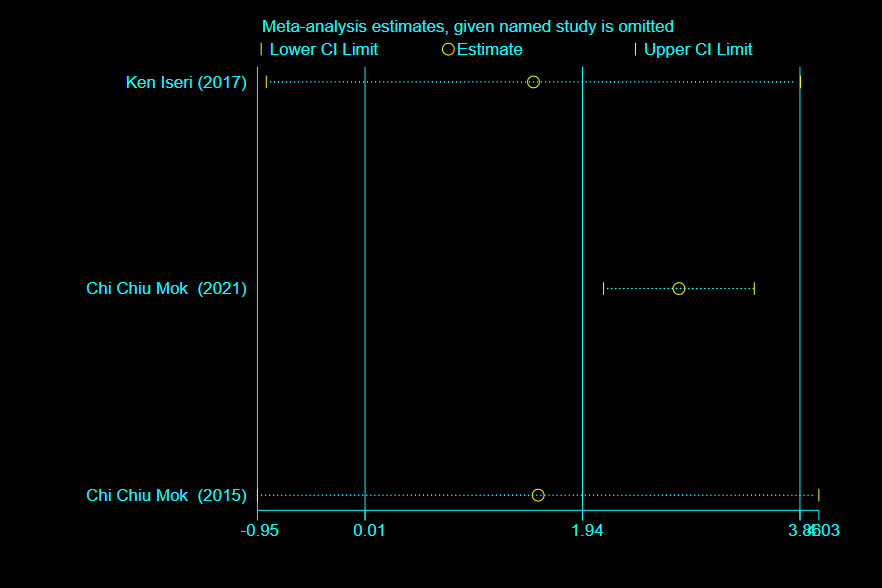


Supplementary Figure 3：Sensitivity analysis of denosumab-associated total hip bone mineral density (BMD) change.
